# Serotonergic Modulators in Alzheimer's Disease: A Hope in the Hopeless Condition

**DOI:** 10.1002/cbdv.202403401

**Published:** 2025-06-03

**Authors:** Najlaa Hamed Almohmadi, Hayder M. Al‐Kuraishy, Ali I. Al‐Gareeb, Ali K. Albuhadily, Morkoss M. Fakhry, Athanasios Alexiou, Marios Papadakis, Gaber El‐Saber Batiha

**Affiliations:** ^1^ Clinical Nutrition Department College of Applied Medical Sciences Umm Al‐Qura University Makkah Saudi Arabia; ^2^ Department of Clinical Pharmacology and Medicine College of Medicine Mustansiriyah University Baghdad Iraq; ^3^ Jabir Ibn Hayyan Medical University Kufa Iraq; ^4^ Department of Biochemistry and Molecular Biology Faculty of Pharmacy Egyptian Russian University Cairo Egypt; ^5^ University Centre for Research & Development Chandigarh University Chandigarh‐Ludhiana Highway Mohali Punjab India; ^6^ Department of Research & Development Funogen Athens Greece; ^7^ University Hospital Witten‐Herdecke University of Witten‐Herdecke Wuppertal Germany; ^8^ Department of Pharmacology and Therapeutics Faculty of Veterinary Medicine Damanhour University Damanhour Egypt

**Keywords:** Alzheimer's disease, serotonin, 5‐HT receptors

## Abstract

Alzheimer's disease (AD) is the main cause of dementia worldwide. AD is a progressive brain neurodegenerative disease due to genetic and environmental factors that induce a progressive accumulation of intracellular hyperphosphorylated tau protein and extracellular amyloid protein (Aβ). However, anti‐AD medications cannot reverse the fundamental AD neuropathology due to amyloid plaques and related oxidative stress and inflammatory reactions. Thus, targeting other pathways might be reasonable in the management of AD. The serotonin (5‐HT) neurotransmitter plays a crucial role in preventing neurodegeneration and related oxidative stress and inflammatory reactions. In addition, the serotonergic system is highly dysregulated in many neurodegenerative diseases, including AD. Deregulation of serotonin synthesis and its receptors is involved in the pathogenesis of AD. Therefore, this review aims to discuss how the serotonergic system is affected in AD, and how 5‐HT modulators can reverse AD neuropathology and alleviate the associated neuropsychiatric disorders in AD patients.

## Graphical abstract

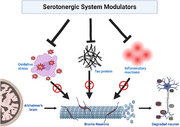

**Abbreviations**
α‐Syn: alpha synuclein
Aβ: amyloid beta
AD: Alzheimer's disease
ApoE4: apolipoprotein A4
AKT/PKB: protein kinase B
AMPK: AMP‐activated protein kinase
APP: amyloid precursor protein
CIP2A: cancerous inhibitor of PP2A
EOAD: early‐onset AD
GSK3β: glycogen synthase kinase 3β
HMG‐CoA: hydroxyl methyl‐gutaryl coenzyme A
LOAD: late‐onset AD
MPTP: 1‐methyl‐4‐phenyl‐1,2,3,6‐tetrahydropyridine
mTOR: mammalian target of rapamycin
PD: Parkinson disease
PI3K: phosphatidylinositol 3‐kinase
PP2A: protein phosphatase 2A
PTEN: phosphatase and tensin homologue
ROS: reactive oxygen species
SNpc: substantia nigra pars compacta

## 1 Introduction

**FIGURE 1:**
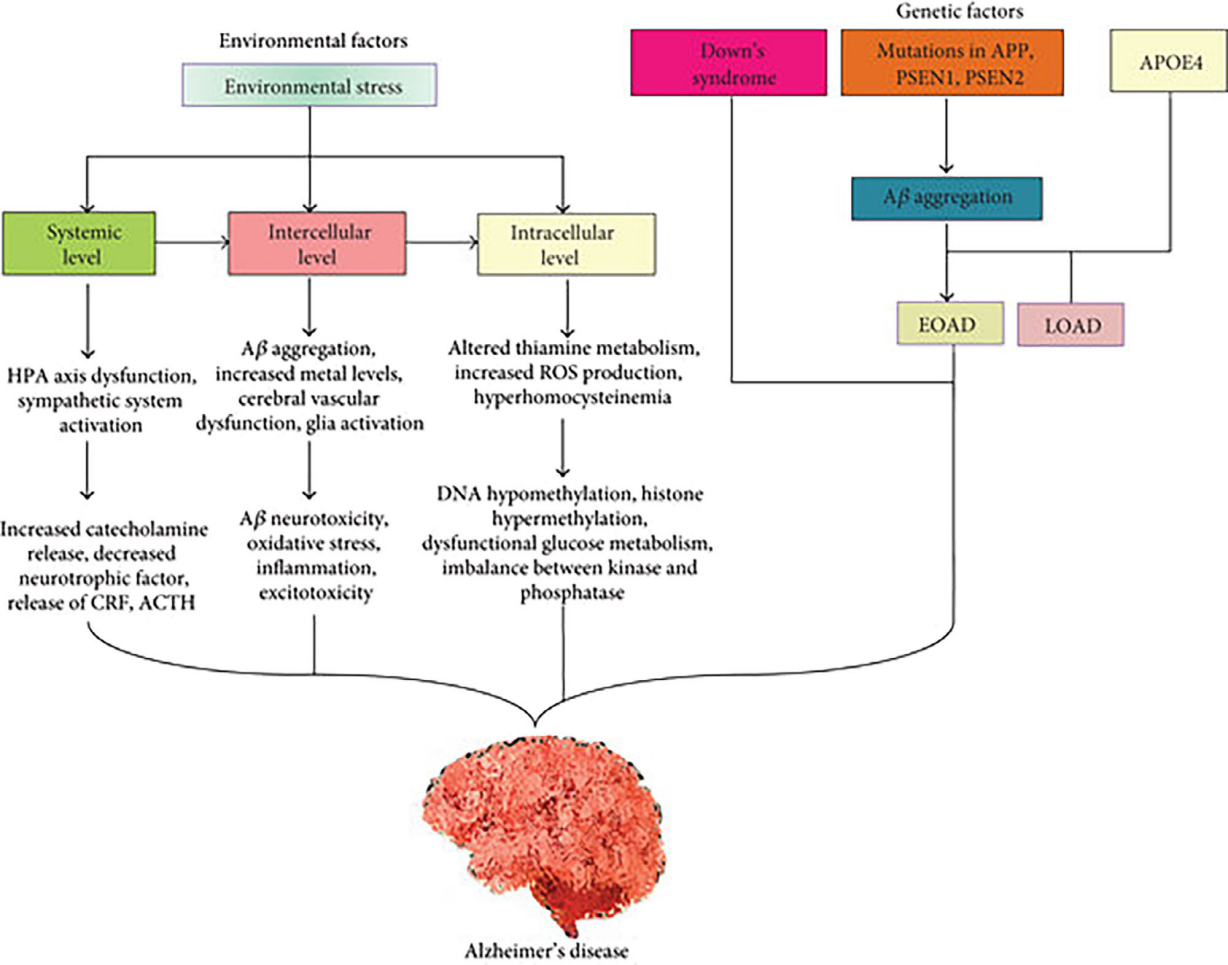
Pathophysiology of AD [151].

Alzheimer's disease (AD) is considered the main cause of dementia in the elderly population around the world [1]. AD is a progressive brain neurodegenerative disease due to genetic and environmental factors that induce a progressive accumulation of intracellular hyperphosphorylated tau protein and extracellular amyloid protein (Aβ) [2] (Figure 1). The advanced deposition of hyperphosphorylated tau protein promotes the formation of neurofibrillary tangles (NFTs); however, the extracellular deposition of Aβ induces the formation of amyloid plaques. Both NFTs and amyloid plaques trigger direct neurodegeneration or indirectly by activating the release of reactive oxygen species (ROS) and proinflammatory cytokines, which induce neuronal apoptosis [3, 4]. A majority of AD cases manifest as a late‐onset sporadic form, but genetically, the disease is divided into familial cases and sporadic cases. The familial form is due to mutations in three major genes (amyloid precursor protein [*APP*] gene, presenilin1 [*PSEN1*] gene, and presenilin 2 [*PSEN2*] gene). Familial AD, which represents 5%–10% of all AD cases, is linked with the development and progression of early‐onset AD (EOAD). However, sporadic AD, the most common type, represents 90% of all AD cases and contributes to the development of late‐onset AD (LOAD) [5, 6]. Familial AD is mainly related to the overproduction of Aβ; however, sporadic AD is mainly related to the defect in the clearance of Aβ [7, 8]. Interestingly, apolipoprotein A4 (ApoE4), which is involved in the regulation of brain cholesterol metabolism, is involved in the pathogenesis of EOAD and LOAD [9]. These neuropathological disorders involved in AD neuropathology distort synaptic plasticity and the release of different neurotransmitters [10]. Particularly, cholinergic neurons in the prefrontal cortex and hippocampus are mainly affected in AD, resulting in cognitive impairment and memory dysfunction [11]. Therefore, restoration of cholinergic neurotransmission by cholinergic agonists such as tacrine and donepezil could be effective in the management of AD [12]. Tacrine is a drug used in the treatment of AD as a cognitive enhancer and inhibitor of the enzyme acetylcholinesterase (AChE). However, its clinical application has been restricted due to its poor therapeutic efficacy and high prevalence of detrimental effects [12]. However, these anti‐AD medications cannot reverse the fundamental AD neuropathology due to NFTs, amyloid plaques, and related oxidative stress and inflammatory reactions [13]. Thus, targeting other pathways might be sensible in the management of AD, principally in the alleviation of NFTs and amyloid plaques and related reactions.

The serotonin (5‐HT) neurotransmitter plays a crucial role in preventing neurodegeneration and related oxidative stress and inflammatory reactions [14]. In addition, the serotonergic system is highly dysregulated in many neurodegenerative diseases, including AD [15]. Impairment of serotonin synthesis and the expression of 5‐HT receptors are implicated in the pathogenesis of AD [16]. Therefore, this review aims to discuss and explain how the serotonergic system is affected in AD, and how 5‐HT receptor agonists can reverse AD neuropathology and alleviate the associated neuropsychiatric disorders in AD patients.

## 2 Serotonin System: An Overview

**FIGURE 2:**
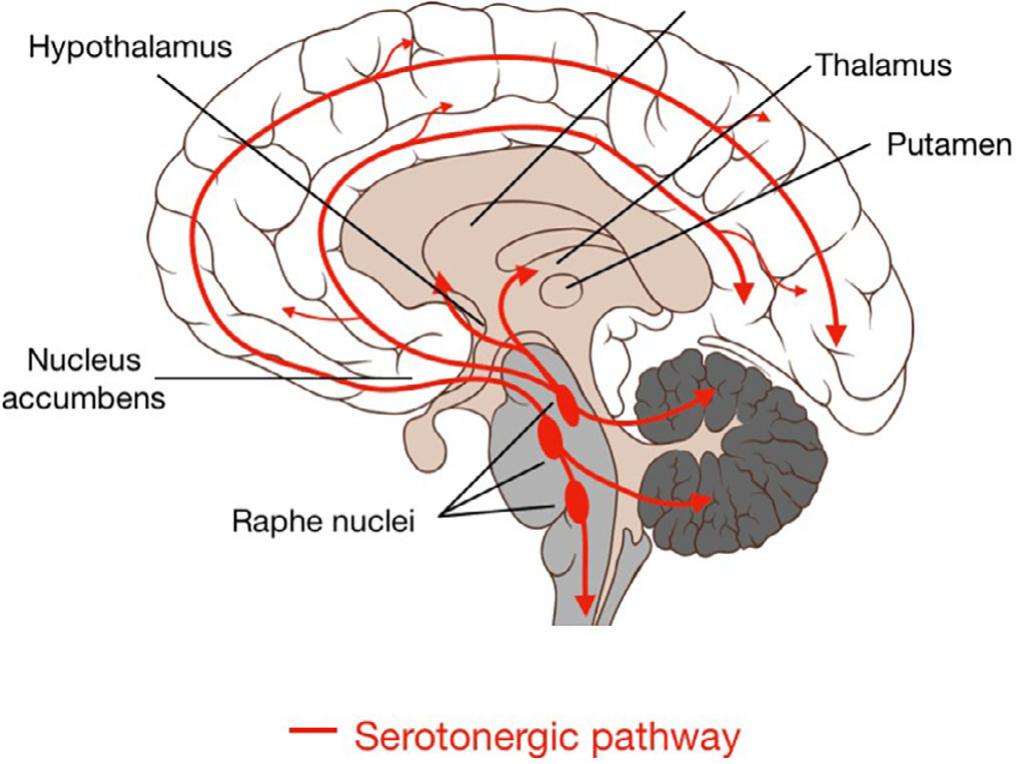
Distribution of the 5‐HT system in the brain [152].

Serotonin or 5‐hydroxytryptamine (5‐HT) is a well‐known monoamine neurotransmitter involved in diverse biological functions [17]. 5‐HT was initially discovered in 1948 by Maurice M. Rapport and his coworkers at the Cleveland Clinic as a vasoconstrictor substance known as serotonin [18]. 5‐HT is involved in the regulation of mood, memory, learning, and sexual function [19]. In addition, 5‐HT has many peripheral functions such as vasoconstriction and platelet aggregation. Approximately 90% of 5‐HT in the human body is produced by enterochromaffin cells of the gastrointestinal tract, 8% by platelets and 2% in the central nervous system (CNS) [20, 21]. 5‐HT in the CNS is mainly located in the raphe nuclei, which are located in the reticular formation of the brainstem [22] (Figure 2). 5‐HT from enterochromaffin cells is released into the circulation, where it is actively uptaken by platelets [23]. Under physiological conditions, released 5‐HT from platelets induces vasodilation by inducing the release of nitric oxide (NO) and inhibiting the release of norepinephrine [24]. However, during endothelial injury, platelet 5‐HT triggers vasoconstriction by contracting vascular smooth muscles [25]. Therefore, 5‐HT dysregulation is implicated in the pathogenesis of hypertension and atherosclerosis [26].

Of note, 5‐HT is synthesized from the amino acid tryptophan by tryptophan hydroxylase, which forms l‐5‐hydroxytryptophan, which is converted by aromatic l‐amino acid decarboxylase to 5‐HT [27]. In addition, 5‐HT hydroxylase catalyzes 5‐HT neurotransmitter synthesis in the CNS and the periphery [27]. 5‐HT is metabolized by monoamine oxidase (MAO) to 5‐hydroxyindolacetic acid (5‐HIAA) or stored by presynaptic vesicles [28]. The released 5‐HT in response to the action potential induces excitatory effects via activation of post‐synaptic 5‐HT receptors and controls the release of 5‐HT through activation of presynaptic receptors [29]. Furthermore, 5‐HT in the synaptic cleft is uptaken by special monoamine transporters into the presynaptic neurons [30].

5‐HT acts by activating 5‐HT receptors (Table 1), which are expressed on neurons and other cells. There are seven types of 5‐HT receptors (5‐HT1–5‐HT7), all are G‐protein coupled receptors except 5‐HT3 which is a ligand ion channel [29, 31, 32]. Importantly, the 5‐HT system is a target site for several drugs such as antidepressants, anxiolytics, and antipsychotics [33].

**TABLE 1:** List of 5‐HT receptors involved in AD disorders.

5‐HT receptor subtype	Role in Alzheimer's disease	Pathophysiological implications	Potential therapeutic targets	References
5‐HT_1A_	‐ Critical for memory and learning ‐ Dysregulation linked to cognitive deficits and neuropsychiatric symptoms (anxiety, depression)	‐ Desensitization impairs serotonergic signaling ‐ Loss of serotonergic neurons correlates with neuroinflammation and tau hyperphosphorylation	‐ SSRIs may alleviate neuropsychiatric symptoms	[116, 117, 118]
5‐HT_1B_	‐ Involved in modulating neurotransmitter release ‐ Reduced density linked to cognitive decline	‐ Impaired cholinergic activity affects memory processes	‐ Antagonists may help manage non‐cognitive symptoms	[119, 120]
5‐HT_1D_	‐ Primarily located presynaptically ‐ Plays a role in inhibiting neurotransmitter release	‐ Dysregulation may lead to altered serotonergic signaling and mood disorders	‐ Selective agonists could enhance serotonergic function	[121, 122]
5‐HT_1F_	‐ Modulates neurotransmitter release in the CNS ‐ Potential implications for migraine treatment	‐ Altered signaling may affect pain perception and mood disorders	‐ Selective agonists may provide migraine relief	[123, 124, 125]
5‐HT_2A_	‐ Involved in cognitive functions and perception ‐ Dysregulation linked to psychotic symptoms in AD	‐ Associated with neuroinflammation and cognitive decline	‐ Antagonists may be beneficial for treating schizophrenia and AD‐related psychosis	[126, 127]
5‐HT_2B_	‐ Linked to cardiovascular function and gastrointestinal motility ‐ Role in mood regulation is being explored	‐ Potential involvement in behavioral changes associated with AD	‐ Antagonism may help manage gastrointestinal issues	[128, 129, 130]
5‐HT_3_	‐ Ligand‐gated ion channel involved in fast synaptic transmission ‐ Associated with nausea and anxiety regulation	‐ Altered function may contribute to anxiety and cognitive disturbances in AD	‐ Antagonists are used to prevent chemotherapy‐induced nausea	[131, 132, 133]
5‐HT_4_	‐ Promotes nonamyloidogenic processing of APP ‐ Neuroprotective effects against amyloid pathology	‐ Increases levels of soluble APPα (sAPPα), which has neuroprotective properties	‐ Agonists could mitigate amyloid‐β accumulation	[134, 135, 136]
5‐HT_5A_	‐ Less understood; potential involvement in mood regulation and cognition	‐ Limited data on its role in AD; further research needed	‐ Future studies required for therapeutic implications	[137, 138]
5‐HT_6_	‐ Associated with cognitive impairment and behavioral disturbances (aggression, psychosis)	‐ Reduced receptor density correlates with cognitive deficits and behavioral changes in AD patients	‐ Antagonists may benefit cognitive functions and behavioral symptoms	[139, 140, 141]
5‐HT_7_	‐ Involved in psychotic symptoms (hallucinations, behavioral changes observed in AD patients	‐ Decreased mRNA levels correlate with psychotic symptoms	‐ Selective antagonists may manage psychotic symptoms effectively	[142, 143, 144]

## 3 Role of 5‐HT in AD

Mounting evidence indicates that 5‐HT can regulate neuronal proliferation, differentiation, migration, and apoptosis [34]. In addition, the serotonergic system regulates the production and clearance of misfolded proteins, including tau protein and Aβ [34, 35]. It has been shown that 5‐HT is reduced and associated with synaptic dysfunction in AD [36]. Therefore, 5‐HT, as it improves memory and cognitive functions, is regarded as a therapeutic target in the management of AD [37]. A case‐control study observed that the hippocampal density of 5‐HT1 was reduced by 24% in patients with mild cognitive impairment, and by 49% in AD patients compared to healthy controls [38]. Consistently, a previous case‐control study illustrated that 5‐HT‐producing neurons in the raphe nuclei were significantly reduced compared to healthy controls. Importantly, injury of raphe nuclei and loss of 5‐HT‐producing neurons are correlated with the severity of symptoms in AD patients [39].

It has been revealed that a reduction in the expression of 5‐HT receptors is correlated with the development of depression in AD patients [40]. Of note, polymorphisms of 5‐HT_2A_ and 5‐HT_2C_ augment the risk of depression fivefold in patients with LOAD compared to healthy controls [41]. Thus, restoration of serotonergic signaling by selective serotonin‐reuptake inhibitors (SSRIs) could improve depressive symptoms and mitigate AD neuropathology [42].

The underlying cause for the reduction of 5‐HT levels and the expression of the 5‐HT receptor is related to AD neuropathology. It has been confirmed that Aβ and associated oxidative stress and neuroinflammation augment the expression of serotonin transporters (SERTs) in transgenic mice [43, 44]. SERTs decrease the concentration of 5‐HT in the extracellular and synaptic cleft. Noristani et al. [45] found that higher concentrations of Aβ and tau protein promote the expression of SERTs in the hippocampus of transgenic mice with EOAD. However, in the LOAD with progressive neurodegeneration, the expression of SERTs is extremely reduced [46]. Furthermore, chronic low‐grade inflammation and the release of proinflammatory cytokines due to microglia activation in AD promote the development of neuroinflammation [47]. Interestingly, proinflammatory cytokines, mainly IL‐1β and TNF‐α, promote the expression of SERTs [48]. Therefore, tau protein, Aβ, and associated inflammation might be a possible mechanism for the deregulation of serotonergic neurotransmission in AD.

Activation of the 5‐HT system attenuates the aggregation of Aβ in the brain. Administration of antidepressant SSRIs reduces Aβ in brain interstitial fluid by 25%. However, chronic treatments with SSRIs decrease Aβ in the brain interstitial fluid by 50%. Similarly, direct administration of 5‐HT into the hippocampus prevents the production and accumulation of Aβ in transgenic mouse models. Consistently, findings from retrospective studies illustrated that prolonged use of SSRIs > 5 years reduced brain Aβ load measured by positron‐emitting tomography in depressed patients compared to SSRI nonusers [42, 48–52]. However, there are contradictory findings regarding the effects of SSRIs on AD. For example, von Linstow et al. [53] observed that 5‐HT augmentation therapy by the SSRI escitalopram has minimal effects on Aβ levels in early‐stage AD‐like disease in mice. Remarkably, levels of insoluble Aβ_40_ increased in the neocortex but not in the hippocampus of SSRI‐treated mice compared with those treated with vehicle control, but they were unaffected in the hippocampus [53]. This finding suggests that modulation of the 5‐HTergic system has either no effect or increases Aβ neuropathology. Conversely, an experimental study conducted by Sheline et al. [54] observed that a single ip injection of escitalopram (5 mg/kg/day) reduced the levels of Aβ_40_ in the hippocampal interstitial fluid of APP/PS1 mice. Furthermore, chronic treatment with the SSRI paroxetine did not mitigate Aβ pathology and Aβ plaque‐induced microgliosis in the hippocampus of APP transgenic mice [55]. In addition, 3 months of 5‐HT depletion did not significantly impact the Aβ plaque load or Aβ_42_/Aβ_40_ ratio in APP transgenic mice. Thus, SSRIs treatment may be ineffective in the management of AD. Importantly, despite the fact that paroxetine occupies > 80% of serotonin transporters, it had no effect on the Aβ plaque load, number and size of plaques, and the Aβ plaque‐induced increases in microglial numbers in the dentate gyrus of APP transgenic mice [55]. These findings propose that factors other than dosing should be identified to clarify any discrepancies between different SSRI drugs.

These verdicts indicated that deregulation of 5‐HT synthesis and serotonergic neurotransmission is implicated in the pathogenesis of AD.

## 4 Dysregulation of 5‐HT Receptors in AD

Different studies highlighted that dysregulation of 5‐HT receptors is involved in AD neuropathology and related symptoms. In particular, selective dysregulation of 5‐HT receptors is implicated in AD and the progression of symptom severity. For example, 5‐HT_1A_ is mainly expressed in the presynaptic neurons of the hippocampus that control cognitive and memory functions. 5‐HT_1A_ inhibits the release of 5‐HT; therefore, pharmacological inhibition of this receptor can improve cognitive function in AD [56]. It has been shown that the hippocampal expression of 5‐HT_1A_ is highly reduced in the early stages of AD patients compared to healthy controls [57]. Therefore, modulating the functional activity of 5‐HT_1A_ could be an effective therapeutic strategy in the management of AD (Table 1).

Similarly, 5‐HT_1B_, which controls the release of 5‐HT, is reduced by the effect of APP in transgenic mice. However, the expression of SERTs and MAO are augmented, leading to the reduction of 5‐HT in the AD [58]. Furthermore, 5‐HT_1B_ has a potent anti‐inflammatory effect, and 5‐HT_1B_ agonists could be effective in reducing neuroinflammation in AD. 5‐HT_1B_ agonists prevent the release of proinflammatory cytokines and the inflammatory signaling pathway ERK1/2, which mediate Aβ‐induced neurotoxicity in AD [59]. Recently, 5‐HT_2B_ mRNA expression was widely diverse in adult microglia and was higher compared to other cortical cell subtypes. The density of 5‐HT_2B_ was low and overall reduced in transgenic mice compared to wild‐type mice [60]. Thus, Aβ in AD reduces the affinity of 5‐HT_2B_ binding to 5‐HT, which advocates biased agonists, rather than antagonists, might be useful for AD patients. Also, the expression of 5‐HT_1D_, 5‐HT_1E_, and 5‐HT_1F_ is reduced in the hippocampus of AD [59, 61].

Moreover, 5‐HT_2A_, which regulates the release of dopamine and glutamate in the prefrontal cortex, is extremely dysregulated and is associated with the alteration of dopaminergic neurotransmission in AD (Table 1) [62]. Lu et al. [63] observed that overexpression of the 5‐HT_2A_ receptor promotes AD neuropathology. Consistently, inhibition of the 5‐HT_2A_ receptor by the antihistamine desloratidine attenuates AD neuropathology by inhibiting neuroinflammation in transgenic mice [63]. In addition, 5‐HT_2A_ receptor inverse agonist pimavanserin reduces Aβ‐induced neuropathology [64]. Moreover, the 5‐HT_2B_ receptor, which has a neurotoxic effect, is involved in AD neuropathology by increasing APP processing and the production of brain Aβ. Remarkably, the expression of 5‐HT_2B_ receptors has been reported to be augmented in AD patients compared to healthy controls [65]. In addition, the 5‐HT_2C_ receptor, which reduces dopamine release in the mesolimbic cortex and increases the release of acetylcholine in the prefrontal cortex, is highly dysregulated in the pathogenesis of AD [66]. Activation of serotonergic neurotransmission by 5‐HT_2C_ receptor agonists mitigates cognitive impairment in the AD model [67].

However, the expression of 5‐HT_3_ receptors in neurons promotes the production of Aβ in AD [68], suggesting a neurotoxic effect of this receptor. Consistently, administration of 5‐HT_3_ receptor antagonist for 8 weeks attenuates AD neuropathology in AD [68]. In addition, overexpression of 5‐HT_3_ receptors in the hippocampal GABA interneurons is correlated with cognitive dysfunction and behavioral disorders in AD [69] (Table 1). Moreover, the 5‐HT_4_ receptor has a neuroprotective effect against AD neuropathology by reversing the Aβ neurotoxic effect. Indeed, the 5‐HT_4_ receptor agonist usmarapride attenuates cognitive decline in the AD model [70] (Table 1). Findings from a preclinical study revealed that 5‐HT_4_ agonists reduced Aβ load by activating the non‐amyloidogenic pathway, which produces the neuroprotective soluble APPα instead of Aβ [43]. Likewise, 5‐HT_4_ receptor agonist prucalopride suppresses tauopathy in PS19 transgenic mice [71]. Therefore, activation of the 5‐HT_4_ receptor could be an important rational protocol for the future treatment of AD [70, 71] (Table 1). In addition, 5‐HT_5_ is involved in memory consolidation and has a neuroprotective effect against AD neuropathology by modulating inflammatory signaling pathways (Table 1) [72]. Of interest is that 5‐HT_6_ has a complex function in the regulation of memory and cognitive function by controlling the release of acetylcholine and glutamate in AD [49]. 5‐HT_6_ receptor density is reduced in the cerebral cortex of AD patients and associated neuropsychiatric symptoms [50, 51]. Importantly, 5‐HT_6_ inhibits the release of acetylcholine. Thus, 5‐HT_6_ antagonists have been reported by clinical trials to be effective in the management of AD [52]

Moreover, the 5‐HT_7_ receptor, which regulates cognitive function, is upregulated and associated with the development of psychotic symptoms in AD patients [49]. Thus, 5‐HT_7_ receptor antagonists may be effective in the management of neuropsychiatric symptoms in AD patients. Conversely, the 5‐HT_7_ receptor, which enhances hippocampal long‐term potentiation, can improve cognition in AD. A preclinical study revealed that activation of 5‐HT_7_ receptors improves cognitive function and long‐term potentiation in AD by inhibiting neuronal apoptosis in the hippocampus [73] (Table 1). Hence, the potential effect of the 5‐HT_7_ receptor seems controversial and needs to be verified by future studies.

## 5 Modulation of 5‐HT Neurotransmission in AD

It has been shown that SSRIs such as fluoxetine and sertraline, which are commonly used in the management of depression, have neuroprotective effects against neuropsychiatric disorders in AD [74, 75]. Of interest, depression augments AD risk due to the development of neuroinflammation, oxidative stress, and neuronal apoptosis [76]. A longitudinal study observed that the incidence of AD was higher among patients with severe depression compared to nondepressed patients [76]. Therefore, chronic depression is regarded as a potential risk factor for the development and progression of AD [77]. Thus, early management of depression by antidepressant agents, including SSRIs could be an effective preventive measure against the development of AD (Table 2).

**TABLE 2:** List of 5‐HT modulators involved in AD treatment.

Drug class	Mechanism	Efficacy on cognition	Efficacy on behavioral symptoms	Examples	References
Selective serotonin reuptake inhibitor (SSRIs)	Inhibit serotonin reuptake; increase synaptic serotonin levels	Mixed results; some improvement via depression alleviation	Effective for managing depression; mixed effects on anxiety	Citalopram, escitalopram, fluoxetine, sertraline	[145, 146]
Serotonin agonists	Activate specific serotonin receptors (e.g., 5‐HT_4_ and 5‐HT_6_	Potential for cognitive enhancement; ongoing studies	May improve behavioral symptoms through enhanced neurotransmission	RS 67333 (5‐HT_4_ agonist), SB 399885 (5‐HT_6_ agonist)	[147, 148]
Serotonin antagonists	Block specific serotonin receptors (e.g., 5‐HT_2A_, 5‐HT_7_)	Limited evidence; potential for improvement	Effective for reducing psychotic symptoms; may alleviate anxiety	Clozapine (5‐HT_2A_ antagonists), SB 699551 (5‐HT_7_ antagonists)	[149, 150]

Many studies confirmed that SSRIs reduce AD neuropathology by decreasing the production and the deposition of Aβ and hyperphosphorylated tau protein [78, 79]. Fluoxetine and citalopram reduce Aβ production within 24 h of their administration in animal models. This finding was confirmed by the direct administration of 5‐HT into the hippocampus but not by other non‐SSRIs such as tianeptine [79]. In addition, fluoxetine reduces the production of Aβ_40_ and Aβ_42_, inhibits astrocyte activation and the development of neuroinflammation, improves synaptic plasticity by activating hippocampal neurogenesis, and attenuates the formation of NFTs. These findings indicated that elevated brain 5‐HT by SSRIs can alleviate AD neuropathology and associated cognitive decline. Citalopram reduces the early formation of Aβ rather than clearing the preexisting one in a dose‐dependent effect [80]. As well, citalopram reduces the production of the neurotoxic Aβ by activating the non‐amyloidogenic pathway through stimulation of α‐secretase [79]. Furthermore, citalopram improves synaptic plasticity and augments long‐term potentiation in animal models [81]. Besides, citalopram inhibits microglia and reduces the development of neuroinflammation, a hallmark of AD [82, 83]. Conversely, SSRI paroxetine failed to decrease amyloid plaque and tauopathy in AD, which might be due to the differential occupancy of brain SERTs [84, 85]. Likewise, an SSRI escitalopram, did not reduce brain amyloidosis but can reduce hippocampal tauopathy [86]. These preclinical studies suggest differential effects of SSRIs on AD neuropathology (Table 2).

Besides, clinical studies highlighted the potential effects of SSRIs against the development of AD. A cohort study found that SSRIs delay the conversion of mild cognitive impairment to symptomatic AD [87]. A longitudinal study showed that prolonged use of SSRIs, mainly sertraline, reduced AD significantly compared to SSRIs nonusers and other antidepressant users [88]. Of interest, SSRIs delays the onset of dementia in patients with Down syndrome [89]. It has been established that treatment for 2 years with SSRIs attenuates cortical atrophy and amyloid burden in patients with AD or mild cognitive impairment [90]. However, Bartels et al. [87] found that CSF Aβ_1–42_ levels were not affected by the effects of SSRIs, signifying that SSRIs have anti‐AD effects by another pathway not involved in Aβ neuropathology. Conversely, many clinical studies confirmed that SSRIs reduced CSF Aβ_1–42_ levels in patients with AD or mild cognitive impairment [91, 92]. A systematic review and meta‐analysis revealed that SSRI fluoxetine has an ameliorative effect against cognitive dysfunction and AD development in patients with chronic depression [93].

## 6 Role of 5‐HT Agonists and Antagonists in AD

It has been illustrated that 5‐HT agonists, mainly of 5‐HT_4_ and 5‐HT_6_ receptors, can enhance cholinergic neurotransmission and promote non‐amyloidogenic processing of APP. Activation of 5‐HT_4_ receptors has been linked to improved memory and cognitive functions and reduced Aβ levels in animal models of AD [94, 95]. Ongoing clinical trials evaluating the efficacy of 5‐HT agonists suggest that enhancing the 5‐HT signaling by 5‐HT agonists may improve the behavioral symptoms of AD patients [96] (Table 2).On the other hand, 5‐HT antagonists which block the action of 5‐HT at specific receptors (like 5‐HT_2A_ and 5‐HT_7_) can alleviate psychotic symptoms and improve mood stability in AD patients by reducing neuroinflammation associated with AD pathology [97]. These receptors are implicated in mood regulation and cognitive processes. Furthermore, antagonizing 5‐HT_7_ receptors reduces anxiety and depression while potentially improving cognitive function. However, antagonists of 5‐HT_2A_ receptors reduce hallucinations and delusions in AD patients. In addition, limited evidence suggests that 5‐HT_7_ antagonists could enhance cognitive performance; however, more research is needed to establish their efficacy in AD and associated neuropsychiatric disorders [97, 98].

### 6.1 Buspirone

Buspirone is a partial agonist of the post‐synaptic 5‐HT_1A_ receptor and antagonist of presynaptic dopamine D2–D4 that is used in the management of generalized anxiety disorders [98]. It has been revealed that buspirone improves cognitive impairment by activating 5‐HT_1A_ receptors and increases dopamine release in the prefrontal cortex [99]. Similarly, buspirone attenuates MK‐801‐induced cognitive impairment by antagonizing D3 receptors in mice [100]. The cognitive enhancer effect of buspirone is mediated by activating the release of 5‐HT and dopamine and enhancing hippocampal neurogenesis [101, 102]. In addition, 5‐HT_1A_ receptor agonists enhance brain energy metabolism by antagonizing NMDA receptors in rats [103]. Furthermore, buspirone improves cognitive function in patients with schizophrenia [104]. Therefore, buspirone was suggested to be effective in the management of anxiety and cognitive impairment in AD patients [105]. Consistently, a retrospective study illustrated that buspirone mitigates neuropsychiatric disorders in patients with dementia, mainly AD and mixed dementia [106]. In addition, buspirone enhances adult hippocampal neurogenesis [107, 108], which is highly distorted in AD. Besides, buspirone attenuates rotenone‐induced Parkinson's disease (PD) by inhibiting neuroinflammation [109], a hallmark of AD neuropathology. However, both 5‐HT_1A_ receptor agonists and antagonists can reduce hippocampal oxidative stress in AD [110]. This remarkable dual effect of 5‐HT_1A_ receptor agonists and antagonists is related to the dynamic alterations of 5‐HT_1A_ receptors in the different stages of AD [57]. Therefore, buspirone could be effective in treating AD by restoring serotonergic neurotransmission.

### 6.2 Trazodone

Trazodone is a mixed 5‐HT receptor agonist and antagonist, 5‐HT reuptake inhibitor, adrenergic receptor antagonist, and weak H_1_ receptor antagonist. In particular, trazodone is partial agonist of 5‐HT_1A_ and antagonist of 5‐HT_2A_ and 5‐HT_2B_. Trazodone is effective in the management of depression and anxiety disorders [111]. Trazodone is effective in treating sleep disturbance in AD patients [112]. A prospective study revealed that the use of trazodone for 6 months improves disinhibition and irritability in AD patients but does not enhance cognitive impairment [113]. Long‐term effect of trazodone improves cognitive decline in AD patients [114]. Moreover, the neuroprotective effect of trazodone against AD neuropathology is related to the inhibition of tau protein hyperphosphorylation in AD [115]. Therefore, trazodone, by increasing the 5‐HT neurotransmission and improving synaptic plasticity, could be effective in the management of AD.

Therefore, SSRIs and 5‐HT modulators have differential effects on AD neuropathology and associated neuropsychiatric disorders (Table 2).

## 7 Conclusions

5‐HT neurotransmission is deregulated and associated with the development of cognitive impairment in AD. In addition, dysregulation of 5‐HT receptors adversely affects the development and progression of AD by affecting the processing of Aβ and hyperphosphorylation of tau protein. Interestingly, selective agonists and antagonists of 5‐HT receptors are a promising therapeutic strategy in the management of AD and related oxidative stress and neuroinflammation. Particularly, SSRIs and 5‐HT modulators have neuroprotective effects against AD neuropathology. However, the long‐term effects of SSRIs and serotonin modulators on AD risk need to be verified by clinical trials and large‐scale clinical studies.

## Author Contributions

**Najlaa Hamed Almohmadi and Hayder M. Al‐kuraishy**: conceptualization, data collection, and writing of the manuscript. **Ali I. Al‐Gareeb**: conceptualization, data collection, and writing of the manuscript, writing, supervision and editing of the manuscript. **Ali K. Albuhadily**: conceptualization, data collection, and writing of the manuscript. **Morkoss M. Fakhry**: writing, supervision and editing of the manuscript, graphical abstract. **Athanasios Alexiou and Marios Papadakis**: conceptualization, data collection, and writing of the manuscript. **Gaber El‐Saber Batiha**: writing, supervision and editing of the manuscript. All authors read and approved the final version of the manuscript.

## Conflicts of Interest

The authors declare no conflicts of interest.

## Data Availability

The authors have nothing to report.
